# New Challenge, New Motivation? Goal Orientation Development in Graduates of Higher Track Schools and Their Peers in Vocational Training

**DOI:** 10.3389/fpsyg.2018.01371

**Published:** 2018-08-03

**Authors:** Sarah Becker, Maximilian Pfost, Cordula Artelt

**Affiliations:** ^1^Department of Educational Research, University of Bamberg, Bamberg, Germany; ^2^Bamberg Graduate School of Social Sciences, Bamberg, Germany; ^3^Leibniz Institute for Educational Trajectories (LG), Bamberg, Germany

**Keywords:** transition to university, vocational training, development of goal orientation, stage-environment fit, latent change analyses

## Abstract

Many studies have demonstrated a decrease in mastery-approach goals and an increase in performance-approach goals after students’ transition from primary to secondary education. A theoretical explanation for this phenomenon is a deteriorating fit between a learner’s needs and environmental conditions. The purpose of this study was to further examine the development of students’ goal orientation after they graduated from a higher track secondary school and transitioned to university or vocational training as compared with peers who chose vocational training earlier. We also examined the fit between the students’ needs and the conditions in the new educational context to elaborate on the differential fit hypothesis. Data from 487 students and trainees who participated in a German longitudinal school study were available for our analyses. Latent change score models indicated a significant increase in mastery-approach and a decrease in performance-approach goals for higher track graduates after they transitioned to a new educational context, paralleled by an adequate fit between the learners’ needs and the new educational context. For their peers who started vocational training early, mastery-approach goals seem to remain stable, whereas performance-approach goals decreased over time. The results are discussed in the context of the stage-environment fit theory.

## Introduction

Pursuing Nicholls (1984) idea of different types of achievement goal orientations, Dweck developed the first broad theory on goal orientation in Dweck (1986), which became prominent in research in different educational contexts. Beside the question of the impact of goal orientation on socioemotional outcomes (e.g., Hulleman et al., 2008; Huang, 2011; Tuominen-Soini et al., 2012) and academic performance (e.g., Greene and Miller, 1996; Elliot and Church, 1997), research has also focused on the development of goal orientation itself. One of the most empirically well-documented key findings is a decrease in mastery-goal (the aim of developing one’s own competencies and skills as well as learning new things) and an increase in performance-goal orientation (the aim of demonstrating one’s own competencies and skills) after the transition from primary to secondary school (e.g., Anderman and Midgley, 1997; Anderman et al., 2002; Shim et al., 2008).

**FIGURE 1:**
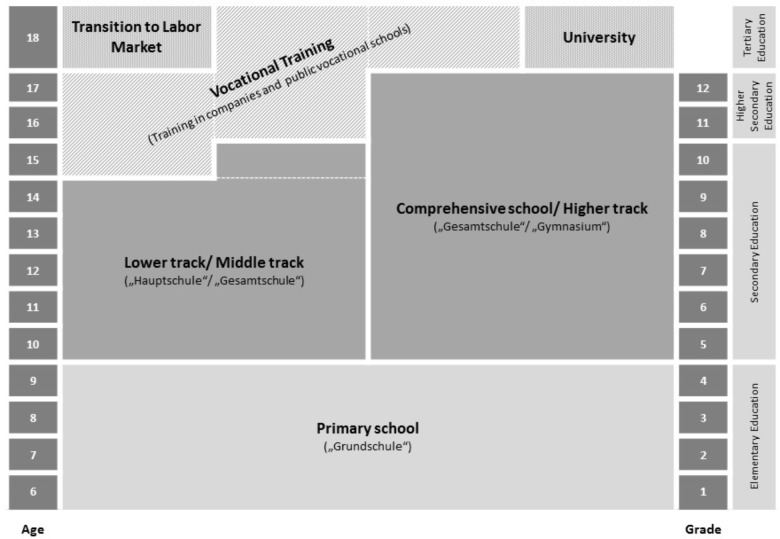
The German school system and the transition to vocational training and university. Comprehensive schools offer all types of leaving certificates.

There are different explanations for the observed changes in goal orientation after the transition from primary to secondary school. One likely explanation was offered by Eccles et al.’s (1993a) stage-environment fit theory. This theory claims that an increasing misfit between a learner’s needs and the learning environment leads to a deterioration in mastery-goal orientation after the transition from primary to secondary school. Therefore, stage-environment fit theory provides a good explanation for the decrease in mastery goal orientation. However, an increase in performance-goal orientation is not well explained by the stage-environment fit theory. Instead, the changes in performance-goal orientation seem to be better explained by the theory of goal structures. Goal structures provide a theoretical framework describing different teaching practices and the learning atmosphere as either mastery or performance oriented (e.g., Roeser et al., 1996). After the transition from primary to secondary school, the learning atmosphere tends to become more performance oriented (Maehr and Zusho, 2009), which explains the increase in students’ performance-goal orientation after the transition to secondary school (e.g., Kaplan et al., 2002). In summary, the stage-environment-fit theory as well as the theoretical framework of goal structures provide a good answer to the question of why changes in students’ motivation after transitioning from primary to secondary school can be observed. However, there is less research on the further development of goal orientation and the question whether these next transitions affect students’ goal orientations, as well as whether these changes occur in accordance with the implications set out by the stage-environment fit theory and goal structures. In an initial study by our research group (Becker et al., 2017), using data from the Bamberg BiKS longitudinal study, we examined the development of students’ goal orientation during the transition from secondary school to higher secondary education (Grades 11 and 12) or to vocational training (**Figure 1**). After this transition, we found an increase in mastery goal orientation, which was higher for the students who began vocational training. We assumed that this increase was an effect of better stage-environment fit and changes in the goal structure of the new learning environment. Changes in the goal structures are especially apparent in German vocational training, which emphasizes the development of subject-specific interests and their practical applications (Weigel et al., 2007). In the framework of goals structures, this could be seen as a rather mastery-oriented learning environment. In the current study, we examined whether the effect of increasing mastery goal orientation could also be found 2 years later for students who graduate from academic track schools and enroll at university or begin vocational training and whether and to what extent performance goal orientation might change either.

### Goal Orientation and Its Impact on Learning Situations

Goal orientation theory concerns the questions of how and why individuals behave in certain ways in different learning and performance situations. After three decades of research on this topic, researchers proposed a trichotomous model that has found solid empirical support (Elliot and Church, 1997; Middleton and Midgley, 1997). The model differentiates between mastery goals, performance-approach goals and a new component: performance-avoidance goals (the aim to hide one’s lack of skills and competencies).

Later, Elliot and McGregor (2001) also integrated the approach-avoidance distinction for mastery goals (the aim to avoid losing one’s own competencies and skills). The approach-avoidance distinction is important because of the differential impact of approach versus avoidance goal orientation on socioemotional outcomes and academic performance. Many studies have postulated the adaptive pattern of mastery-approach goals in learning situations. For example, they have been found to be positively associated with intrinsic motivation (Spinath and Steinmayr, 2012), positive emotions or well-being (Maehr and Zusho, 2009; Huang, 2011), help-seeking behavior (Butler and Neuman, 1995), as well as academic achievement (Greene and Miller, 1996). In contrast, performance-avoidance or mastery-avoidance goals have almost always shown maladaptive correlations for individuals. Mainly performance-avoidance-oriented individuals have shown higher levels of neuroticism (McCabe et al., 2013) and lower performance in education (Elliot and Church, 1997). Mastery-avoidance oriented students, for example, had high values of fear of failure or low self-determination (Elliot and McGregor, 2001). For performance-approach goals, the research has been more diverse. On the one hand, strong positive correlations with academic achievement were found (Harackiewicz et al., 2002), but on the other hand, correlations with high levels of neuroticism were also found (McCabe et al., 2013).

### Development of Goal Orientation During Educational Transitions and Stage-Environment Fit

Numerous studies have shown substantial stability as well as situational variability in goal orientation (Fryer and Elliot, 2007; Husemann et al., 2007; Praetorius et al., 2014). Transitions into new contexts also seem to have a particularly strong influence. On the one hand, the decline in mastery goals after the transition from primary to secondary schools has been well-documented (e.g., Anderman and Midgley, 1997; Anderman and Anderman, 1999). On the other hand, authors have also reported an increase in performance goals. One possible explanation for this development is that changes in contextual conditions result in a misfit between a learner’s needs and the learning environment. Eccles et al. (1993b) described these findings in the context of the stage-environment fit theory. They postulated that a misfit between internal needs (e.g., a need to participate) and environmental conditions leads to a decrease in learning motivation (for an overview see also Eccles, 2004). Research on changes in environmental conditions has shown, for example, a decrease in participation opportunities for students in secondary schools (Eccles et al., 1993b) and deterioration in the quality of the student–teacher relationship (Midgley et al., 2002). Another explanation for the increase in performance orientation are the changes in goal structures that arise after the transition to secondary school. In comparison to primary schools, teachers in secondary education, for example, focus more on performance comparisons due to regulated grading practices with a social comparison norm and feedback which could lead to a more performance-oriented classroom environment (e.g., Köller, 2000; Anderman et al., 2002; Maehr and Zusho, 2009).

Little documentation can be found on the further development of goal orientation and its relation to educational transitions beyond secondary school and to stage-environment fit. Pajares and Cheong (2003), for example, found an increase in mastery-goal orientation after the transition from middle school to high school, whereas performance goals remained stable. Maier and Brunstein (2001) also reported an increase in mastery goal orientation after the transition from secondary education to different educational contexts (university, higher secondary education, or vocational training). A possible explanation for the positive development of mastery goal orientation reported in both studies was again the increase in stage-environment fit or changes in the goal structure of the learning environment: after finishing high school, students’ choice of the next step in their career (e.g., university or vocational training) was found to be more in accordance with their own needs, talents, or interests, leading to a better fit between learner and environment. In this context, the self-determination theory of Deci and Ryan (1985, 2000) can also be considered as a possible explanation. The SDT assumes that people are more intrinsically motivated when their basic needs for “autonomy,” “competence,” and “relatedness” are satisfied. After finishing high school, students can choose their next career steps more autonomously and in correspondence with their specific interests and competencies. The satisfaction of the mentioned needs could be seen as a predictor of mastery goal orientation as, e.g., Janke et al. (2015) have proven empirically in a study with teachers.

This finding was also supported by Vasalampi et al. (2010), who reported an increase in intrinsic reasons for goal striving, as a comparable motivational measure to goal orientations, when such intrinsic reasons were congruent with the skills needed in the transition to a vocational or academic track in Finland’s post-comprehensive schools. Some other studies have postulated a positive impact of a good stage-environment fit on other socioemotional or motivational outcomes. For example, in a longitudinal study of college students, a better fit was positively correlated with personality consistency, self-esteem, and lower values on neuroticism (Roberts and Robins, 2004). Also, the relationship between job satisfaction and self-efficacy was mediated by higher vocational congruence (Pinquart et al., 2003).

Before we describe the aims of the current study, we would like to provide a brief overview of the German school system and the transition to university or vocational training.

### The German School System, the Transition to University or Vocational Training, and Changes in Goal Orientation

In most German states, students change to secondary school at the age of 10 after completing 4 years of primary education (Cortina et al., 2008). At this point, they are separated according to their academic achievement and parental decisions into three different types of secondary school tracks (**Figure 1**). Students spend 5–6 years in a lower academic track school (“Hauptschule” or “Mittelschule”) or 6 years in a middle academic track school (“Realschule”). Certificates received from lower and middle track schools allow students to begin vocational training. Alternatively, students may choose to switch to higher track school, after completing their track, given that their grades pass defined benchmarks. Higher academic track schools (“Gymnasium”) comprise 8 or 9 years of education and qualify students for university admission (“Abitur”). Students who graduate from higher track schools are also allowed to begin vocational training. Compared with lower track schools, higher track schools focus more on academic learning and are characterized by a high level of cognitive activation (Kunter et al., 2005). In addition, in some German states, there are comprehensive schools (“Gesamtschule”), which include all three academic tracks and offer all types of leaving certificates. **Figure 1** shows the different track schools, the number of years attended, and the students’ ages while attending.

German vocational training, which students can begin after graduating from all types of school, normally takes 2–3 years (Cortina et al., 2008). It is also known as the “dual system” because it includes two different learning locations. The practical part of education is usually located at a craftsperson’s business or a company of some size, whereas the theoretical part is taught in public vocational schools. Approximately 50% of all students who graduate from school enter vocational training (Federal Ministry of Education and Research, 2015). The successful completion of vocational training leads to a certification in a particular field of work.

In a prior study by our research group (Becker et al., 2017), we focused on the development of goal orientation in students who began vocational training or went on to attend higher secondary education after graduating from secondary school (i.e., after completing Grade 10 in Germany). To analyze the stage-environment fit, beside asking about goal orientation, we also asked the trainees for the reasons (internal or external) why they had chosen their field of training. Consistent with prior results, we also found an increase in mastery goals. This increase was higher for students who decided to attend vocational training. Furthermore, most of them reported internal reasons (e.g., interest and talent) for choosing their subject. This might be a predictor for a good stage-environment fit. By contrast, performance-approach goals decreased after the transition to the same extent in both groups.

### Aims of the Study and Research Questions

In the present study, the goal was to examine stability and change in goal orientation by focusing on the transition from a higher track school to university or vocational training. With reference to the stage-environment-fit-theory, we expected an increase in mastery-approach-goals. Furthermore, we expected correlations between good stage-environment fit and mastery goal orientation for students who graduated from a higher track school and transitioned to university or vocational training (hereinafter referred as “graduates”). After finishing high school, a students’ choice of the next step in their career (e.g., university or vocational training) was expected to be more in accordance with their own needs, talents, or interests, possibly leading to a better fit between learner and environment and thus to an increase in mastery-approach goal orientation.

We also examined whether there would be a difference between the graduates who went on to university (hereinafter referred as “graduates at university”) or began vocational training (hereinafter referred as “graduates in vocational training”). Especially for graduates in vocational training, we expected an increase in mastery-approach goals, in accordance with the findings from our prior study (Becker et al., 2017), where an increase has been observed.

Peers who began vocational training (hereinafter referred as “trainees”) in our first study (Becker et al., 2017) were in their last year of training when we collected the data for the current study. As far as we know, no research has been done on the further development of mastery-approach goals during vocational training. Therefore, we are also examining this in the current study without making a prior assumption. Since most of the trainees reported a good stage-environment fit at the beginning of their training and there was no other transition, we assume that there is neither a strong increase nor decrease of mastery-approach goals.

In addition, we were also interested in the further development of performance-approach goals in the three groups (graduates at university, graduates in vocational training, and trainees), without making *a priori* assumptions about their development during the time period of our study.

Finally, we are interested in the association between stage-environment-fit and the two types of goal orientation. Therefore, we used the reasons (more internal vs. more external) for choosing a field of study or vocational training. We would like to point out that this measure can only be seen as a proxy for the stage-environment fit, since we only asked the students by themselves and we did not directly collect information about the new educational environment and the environment’s affordances.

Beside this limitation, we expected a positive association between more internal reasons and mastery-approach goals, as well as a positive association between more external reasons and performance-approach goals.

## Materials and Methods

### Sample and Participants

**FIGURE 2:**
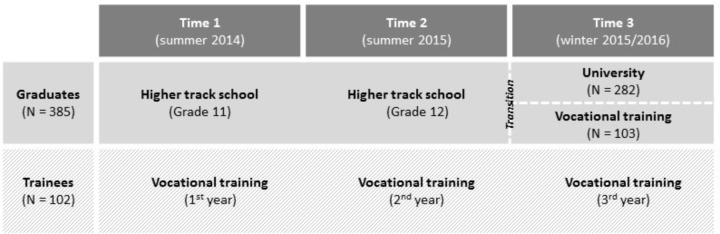
Educational development of the participants of BiKS^plus[8-18]^.

The current data were taken from the Bamberg BiKS^plus[8-18]^ longitudinal study, which is an interdisciplinary research project supported by the German Research Foundation (DFG). BiKS^plus8-18^ is a follow-up of the BiKS study^1^ (for a detailed description of the sample see also: Lorenz et al., 2013), which started in 2006 in southern and central Germany with annual data collection from third-grade students, their parents, and teachers. At the first measurement point (Time 1) of BiKS^plus8-18^ in summer 2014, the subsample (see also **Figure 2**) used in the present study consisted of students who were in Grade 11 in higher track schools (“graduates”) or in their first year of vocational training (“trainees”).

For the analyses, we integrated two additional measurement points. At Time 2 in summer 2015, students were in Grade 12 doing their final higher track school examinations or in their second year of vocational training. At Time 3, in winter 2015/2016, the graduates were in the first year of university or educational training or took a gap year (e.g., social year, language course, internship, traveling, etc.). Most of the trainees were in their last year (third year) of vocational training. As the focus of the analyses was on educational transitions, all students doing a gap year were not considered. The sample consisted of *N* = 385 graduates and *N* = 102 trainees. In the group of 385 graduates, *N* = 282 students started at university, whereas *N* = 103 started vocational training. The average age of all participants was 17.0 years at Time 1 (*SD* = 0.46), 50.3% of all participants were female, and 14.4% had an immigration background.

There were no significant differences between graduates and trainees with respect to age, gender, or immigration background.

### Measures

#### Goal Orientation

We assessed participants’ goal orientation with items from the well-established SELLMO scales (*Skalen zur Erfassung der Lern- und Leistungsmotivation;* Spinath et al., 2002). The authors of SELLMO refer to the trichotomous model of goal orientation and differentiate between mastery-approach (eight items), performance-approach (seven items), and performance-avoidance goals (eight items). In this study, we decided to focus on goals with adaptive patterns, so we included mastery-approach and performance-approach goals in our analyses. Depending on the individual educational context, we used a variation of the item stem to realize the context-specificity (“At university/vocational training/school, it is important to me…”). Item texts were identical across measurement points and contexts. Every item consisted of a statement (e.g., mastery-approach: “At university, it is important to me to get new ideas”; performance-approach: “At university, it is important to me to show that I’m really good at one situation”) that had to be rated on a 5-point Likert scale ranging from 1 (*not at all*) to 5 (*absolutely*). After testing the item characteristics, we excluded one item from each scale in order to avoid scale inconsistency. Internal consistencies were acceptable to satisfactory for both scales at all measurement points (mastery-approach: α_Time1_ = 0.79; α_Time2_ = 0.80; α_Time3_ = 0.79; performance-approach: α_Time1_ = 0.83; α_Time2_ = 0.86; α_Time3_ = 0.84).

#### Reasons for Choosing a Field of Study at University or Vocational Training

In order to get a proxy of the stage environment fit between the learner and the new educational context, we asked the group of graduates at Time 3 why they had chosen their field of study at university or vocational training. To do so, we developed an 11-item questionnaire that included reasons for choosing the subject (e.g., “interest,” “previous experience,” “talent,” “reputation of vocation,” “friends have chosen similar subjects,” etc.). Students had to answer the question “Why did you choose your subject at university/vocational training?” They were then asked to state whether they agreed or disagreed with each reason. In order to assess whether the reasons were internally or externally oriented, the items were later categorized by an expert rating (*N* = 7). To test the interrater-reliability, we calculated the intra-class correlation (ICC = 0.85). Following Wirtz and Caspar’s (2002) recommendation, we identified an ICC > 0.70 as being a good indicator for interrater-reliability. We then chose the reasons that could be clearly assigned to one of the categories by at least four raters. This resulted in three internal (“interest,” “talent,” “previous experience”) and three external reasons (“earning opportunities,” “reputation,” “admission requirement of other subjects are too high”). For the selected reasons, we then constructed two variables (one for internal and one for external reasons) with four categories by summing the number of individual reasons: 0 = *no reason given*, 1 = *one reason given*, 2 = *two reasons given*, 3 = *three reasons given*.

### Data Analyses

**FIGURE 3:**
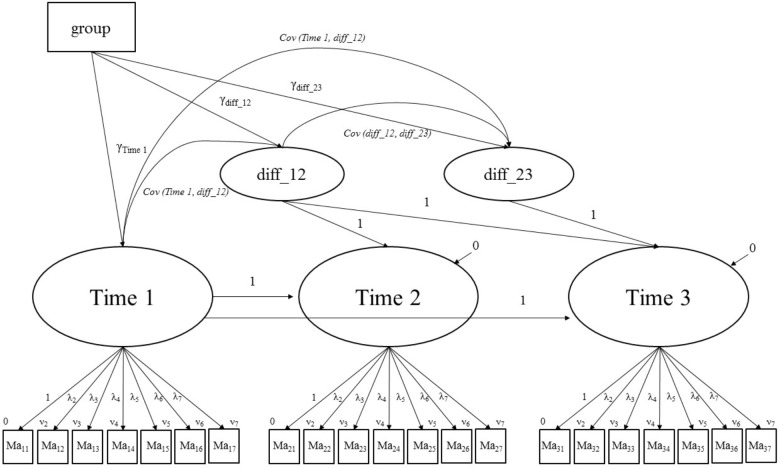
Exemplary Neighbor-Change-Model for mastery-approach goals. Ma_ii_ = Mastery goals: first index describes the measurement point, second index describes the item number; group = dummy-coded group variable (e.g., 0 = trainees; 1 = graduates); diff_ii = difference variables, describe the changes between Times 1 and 2 (diff_12) and Times 2 and 3 (diff_23); not presented: Residual covariances between identically worded items at each measurement point.

We first calculated descriptive statistics for mastery-approach and performance-approach goals for the whole sample and different groups (graduates and trainees) at every measurement occasion. In addition, we computed the means for the predictor of stage-environment-fit measures (internal and external reasons) at Time 3 within the group of graduates. Then we estimated latent change score models for each goal category (also called “Latent Difference Score Models,” McArdle, 2009). In this approach, in contrast to autoregressive models, interindividual differences in intraindividual change are modeled by latent difference variables, which describe the difference between two measurement points by correcting for measurement errors (Geiser, 2010). In neighbor change models (Steyer et al., 1997, 2000), latent difference variables are generated between immediately successive measurement points (**Figure 3**). To analyze our research questions, we chose to use the neighbor change model to interpret the difference between Times 1 and 2 as well as between Times 2 and 3.

Latent change models also allow for the integration of explanatory variables. To examine group differences, we integrated a dummy-coded variable indicating whether a participant was in the group of graduates or trainees (0 = trainees, 1 = graduates). In a second step, in order to clarify whether there were differences within the group of graduates, we calculated the models again with another dummy-coded variable (0 = graduates in vocational training, 1 = graduates at university). A condition that must be fulfilled to calculate changes over time is the comparability of the measurement instruments across the three time points. To ensure this, we used a longitudinal measurement invariance approach. We integrated all three measurement points into one model in which the residuals of the items that were worded in the same way were allowed to covary across time (cf. Little et al., 2007).

We decided to consider the data as categorical as the Kolmogorov–Smirnov test showed that the items neither for the mastery-approach goals nor for the performance-approach goals were normally distributed. Due to the categorical data structure, all measurement models were based on weighted least square means and variance adjusted (WLSMV) estimation. The WLSMV estimator provides robust standard errors so that it can be used for non-normally distributed data and data with skewness or kurtosis (cf. Brown, 2014). The steps of longitudinal measurement invariance with categorical data differ when compared with continuous data because the factor loadings and thresholds must be varied simultaneously (Muthén and Asparouhov, 2002). The parameter restrictions for testing longitudinal measurement invariance for continuous and categorical variables are shown in **Table 1** (cf. Schroeders and Wilhelm, 2011; Edossa et al., 2017).

**Table 1:** Measurement invariance with categorical data.

	Factor	Intercepts	Residual	Factor
	loadings		variances	means
Configural invariance	^∗^	^∗^	Fixed at 1	Fixed at 0
Strong invariance	Fixed	Fixed	Fixed at 1/^∗^	Fixed at 0/^∗^
Strict invariance	Fixed	Fixed	Fixed at 1	Fixed at 0/^∗^


*The asterisk (^∗^) indicates that the parameter is freely estimated. Fixed = the parameter is fixed to equity over time points; Fixed at 1 = residual variances are fixed to 1 at all time points; Fixed at 0 = factor means are fixed at 0 at all time points. Fixed at 0/^∗^ = factor means are fixed at 0 at the first time point and freely estimated at the other time points. Fixed at 1/^∗^ = the residual variances are fixed to 1 at the first time point and freely estimated at the other time points. Parameters in parentheses need to be varied in tandem.*

Following Cheung and Rensvold’s (2002) recommendation, we identified a change in CFI > 0.01 as a serious deterioration in model fit between two consecutive models. As we wanted to compare means between groups, we needed strict measurement invariance.

Finally, we calculated latent regression models to test associations between the predictor for self-reported stage-environment fit (Time 3) and goal orientation (Time 1 and Time 3). All analyses were computed in MPlus 7.3 (Muthén and Muthén, 1998-2015). Missing values were treated by applying a Full Information Maximum Likelihood (FIML) estimator.

## Results

### Descriptive Statistics

**Table 2** shows the means and standard deviations of mastery-approach and performance-approach goals at all measurement points for the whole sample and the subsamples of graduates and trainees. Additionally, values for distribution (skewness and kurtosis) are also reported for the whole sample.

**Table 2:** Means, standard deviation, measures of symmetry, and results of the repeated measures ANOVA of mastery-approach and performance-approach goals.

	Total sample (*N* = 487)				Graduates (*N* = 385)		Trainees (*N* = 102)	
			
	*N*	*M (SD)*	*Skewness*	*Kurtosis*	*N*	*M (SD)*	*N*	*M (SD)*
**Mastery-approach goals**								
Time 1	412	3.94 (0.55)	-0.26	-0.18	322	3.89 (0.54)	90	4.10 (0.53)
Time 2	387	3.89 (0.55)	-0.30	-0.07	311	3.86 (0.53)	76	4.03 (0.62)
Time 3	486	4.14 (0.52)	-0.5	0.25	384	4.17 (0.51)	102	4.05 (0.56)
**Within-subject effects**								
Time	*F*(1, 330) = 6.88; *p* < 0.01							
Time × Group	*F*(1, 330) = 26.53; *p* < 0.01							
**Between-subject effects**								
Group	*F*(1, 330) = 0.66; *p* = 0.42							
**Performance-approach goals**								
Time 1	412	3.25 (0.72)	-0.24	-0.31	322	3.17 (0.71)	90	3.55 (0.64)
Time 2	387	3.00 (0.77)	-0.14	-0.40	311	2.91 (0.76)	76	3.35 (0.70)
Time 3	486	2.99 (0.74)	-0.06	-0.44	384	2.96 (0.74)	102	3.10 (0.75)
**Within-subject effects**								
Time	*F*(1, 330) = 59.18; *p* < 0.01							
Time × Group	*F*(1, 330) = 7.16; *p* < 0.01							
**Between-subject effects**								
Group	*F*(1, 330) = 15.64; *p* < 0.01							



*Time 1: Grade 11 or first year of training; Time 2: Grade 12 or second year of training; Time 3: first year of university/training (graduates) or third year of training (trainees).*

Descriptively mastery-approach goals decreased between Times 1 and 2 in the total sample as well as in the samples of the subgroups. Between Times 2 and 3, there was an increase in mastery-approach goals in the group of graduates. By contrast, the mastery-approach goals in the group of trainees seem to remain more or less stable. The performance-approach goals also seem to decrease between Times 1 and 2 in the total sample and, in addition, between Times 2 and 3 in the group of trainees. In contrast, the mean at Time 3 was a bit higher than the mean at Time 2 in the group of graduates after they transitioned to university or training. In order to strengthen these descriptive results, we reported the results of the latent change models in a later section.

**Table 2** also shows kurtosis and skewness in the two scales for the whole sample. For scale of mastery-approach goals, we found values for skewness and kurtosis between -0.5 and 0.25. The values for performance-approach goals were between -0.44 and -0.06.

**Table 3** compares the means and standard deviations between the students who attended university and the students who started vocational training after graduating from an upper track school.

**Table 3:** Means and standard deviations of mastery-approach and performance-approach goals within the group of graduates.

	Transition to university (*N* = 282)		Transition to vocational training (*N* = 103)	
		
	*N*	*M (SD)*	*N*	*M (SD)*
**Mastery-approach goals**				
Time 1	228	3.91 (0.54)	94	3.87 (0.54)
Time 2	232	3.87 (0.53)	79	3.83 (0.54)
Time 3	282	4.21 (0.49)	102	4.04 (0.53)
**Performance-approach goals**				
Time 1	228	3.17 (0.72)	94	3.18 (0.70)
Time 2	232	2.92 (0.77)	79	2.93 (0.75)
Time 3	282	2.91 (0.71)	102	3.08 (0.80)


*Time 1: Grade 11; Time 2: Grade 12; Time 3: first year of university/training.*

For mastery-approach goals, there were only small group differences between Times 1 and 2. However, the increase between Times 2 and 3 was higher for graduates at university than for graduates in training. We found the same pattern for the development of performance-approach goals. There were also only small group differences between Times 1 and 2. Whereas the group of graduates at university showed almost no change between Times 2 and 3, we found a small increase for graduates in training.

A correlation matrix of Time 1, Time 2 and Time 3 for the subsamples of graduates and trainees can be found in the Supplementary Table 1.

We then analyzed the reasons for choosing a field of study or vocational training in the group of graduates at Time 3 (first year of university or first year of training). The frequencies for the three internal reasons were: “interest” = 98.8%, “talent” = 93.3%, and “previous experience” = 51.0%. In comparison, the external reasons were generally stated less frequently: “earning opportunities” = 53.4%, “reputation” = 38.9%, and “admission requirements of other subjects are too high” = 15.9%. The results indicated that graduates stated more internal than external reasons for choosing a field of study or vocational training. The analysis of the two constructed variables for internal and external reasons showed that graduates on average stated *M* = 2.39 (*SD* = 0.63) internal and *M* = 1.10 (*SD* = 0.87) external reasons.

### Longitudinal Measurement Invariance

We tested configural, strong, and strict longitudinal measurement invariance separately for mastery-approach goals and performance-approach goals with increasing model constraints (see also **Table 1**). To evaluate model deterioration, we focused on the CFI and RMSEA because the χ^2^ statistic is overly sensitive when the sample size is large (Steenkamp and Baumgartner, 1998). We used the DIFFTEST option, which allowed us to test for nested model fit with the WLSMV estimator (Muthén and Muthén, 1998-2015) for categorical data. **Table 4** displays the results of the different models and the changes in model fit. The results indicated strict longitudinal measurement invariance across the three time points for both mastery-approach goals and performance-approach goals. These results enable a meaningful interpretation of the following latent change models.

**Table 4:** Longitudinal measurement invariance for mastery-approach and performance-approach goals.

	χ^2^ *(df)*	CFI	RMSEA		Δχ^2^ *(*Δ*df)*^1^	ΔCFI	ΔRMSEA
**Mastery-approach goals**							
Configural	351.69 (165)	0.962	0.048				
Strong	420.51 (192)	0.956	0.047	M1 vs. M2	82.25 (39)	-0.006	0.001
Strict	431.92 (218)	0.957	0.045	M2 vs. M3	27.52 (14)	0.001	-0.002
**Performance-approach goals**							
Configural	596.85 (114)	0.942	0.093				
Strong	655.85 (155)	0.940	0.081	M1 vs. M2	88.713 (41)	-0.002	-0.012
Strict	649.42 (165)	0.942	0.078	M2 vs. M3	28.740 (10)	0.002	-0.003


*^1^Differences in χ^2^ are based on DIFFTEST function for WLSMV estimator and can therefore not be calculated on the basis of the presented χ^2^-values.*

### Latent Change Models for Mastery-Approach and Performance-Approach Goals

In order to examine the changes in goal orientation over the three time points, and to differentiate between the three groups, we present the results of the latent change models separately for mastery-approach and performance-approach goals. To be able to integrate the dummy-coded group variable based on categorical data into our models, we first had to make sure that the patterns of answer categories were comparable between the groups. For this reason, we had to collapse the answer categories of the goal-orientation items that no one had chosen. We present standardized model results (STDYX standardization).

The model for mastery-approach goals showed good fit (χ^2^ = 523.72, *df* = 236, *p* < 0.01; CFI = 0.942; RMSEA = 0.050). The latent mean of Time 1 for the group of trainees was *M*_Time1_ = 3.73 (*p* < 0.01). The difference variable *diff_12*, which describes the change in mastery-approach goals between Time 1 and Time 2, had a mean of *M*_diff_12_ = -0.16 (*p* = 0.37). The negative value showed a weak decrease in mastery-approach goals between the first and second years of training, but it was not significant. The difference variable *diff_23* describes the changes between the second and third years of training and had a mean of *M*_diff23_ = 0.05 (*p* = 0.966). In summary, there was no significant change in mastery-approach goals during the vocational training. Taking into account the dummy-coded group variable (0 = trainees, 1 = graduates), we found the following group differences at Time 1 (γ_Time1_ = -0.16, *p* < 0.01). Graduates had significant lower values on mastery-approach goals. The regression coefficient of the first difference variable was not significant (γ_diff_12_ = 0.02, *p* = 0.81), showing that there was no group difference in the change between Times 1 and 2. Mastery-approach goals also remained stable for the group of graduates while they were still attending a higher track school. By contrast, the regression coefficient of the second difference variable *diff_23* was positive and significant (γ_diff_12_ = 0.29, *p* < 0.01). Graduates showed a significantly larger increase in mastery-approach goals during their transition to university or vocational training in comparison with trainees during their vocational training (second to third year). All findings are summarized in **Table 5**.

**Table 5:** Latent means, regression coefficients, and covariances for the latent change model for mastery-approach goals.

	Est.	*SE*	Est./*SE*	*p*
**Means**				
*M*_Time_ *1*	3.73	0.29	12.90	0.000
*M*_diff_12_	-0.16	0.17	-0.90	0.370
*M*_diff_23_	0.01	0.12	0.04	0.966
**Regression coefficients (β)**				
γ_Time1_	-0.16	0.05	-3.11	0.002
γ_diff_12_	0.02	0.08	0.24	0.810
γ_diff23_	0.29	0.05	5.56	0.000
**Covariances**				
*Time 1* with *diff_12*	-0.35	0.07	-5.06	0.000
*Time 1* with *diff_23*	-0.15	0.07	-2.08	0.037
*diff_12* with *diff_23*	-0.34	0.08	-4.45	0.000


*Est., estimated parameter; *SE*, standard error; Est./*SE*, ratio of the parameter estimate to the standard error; *p*, significance; regressions coefficients weighted on the dummy-coded group variable (0 = trainees; 1 = graduates); STDYX Standardization.*

The fit for the model for performance-approach goals was acceptable (χ^2^ = 686.24, *df* = 180, *p* < 0.01; CFI = 0.941; RMSEA = 0.076; see also **Table 6**). The latent scale mean at Time 1 for the group of trainees was *M*_Time1_ = 4.05 (*p* < 0.01) and was therefore slightly larger than the mean of mastery-approach goals at the same time. The means of the two difference variables were negative and significant (*M*_diff_12_ = -0.31, *p* < 0.05; *M*_diff_23_ = -0.46, *p* < 0.01), indicating a significant decrease in performance-approach goals across the first and second years as well as the second and third years of vocational training. Adding the group variable, we found a significant regression coefficient at Time 1 (γ_Time1_ = -0.23, *p* < 0.01). Graduates had significant lower values on performance-approach goals at the first measurement point. The regression coefficient of the first difference variable was not significant (γ_diff12_ = -0.06, *p* = 0.35). This indicates stable group differences between Time 1 and Time 2. The regression coefficient of the second difference variable was positive and significant (γ_diff23_ = 0.21, *p* < 0.01), indicating that the decrease between the second and third measurement points was smaller for the group of graduates.

**Table 6:** Latent means, regression coefficients, and covariances for the latent change model for performance-approach goals.

	Est.	*SE*	Est./*SE*	*p*
**Means**				
*M*_Time_ *1*	4.05	0.25	16.40	0.000
*M*_diff_12_	-0.31	0.14	-2.29	0.022
*M*_diff_23_	-0.46	0.12	-3.80	0.000
**Regression coefficients (β)**				
γ_Time1_	-0.23	0.05	-5.02	0.000
γ_diff_12_	-0.06	0.06	-0.93	0.351
γ_diff_23_	0.21	0.06	3.76	0.000
**Covariances**				
*Time 1* with *diff_12*	-0.26	0.06	-4.14	0.000
*Time 1* with *diff_23*	-0.21	0.07	-3.06	0.002
*diff_12* with *diff_23*	-0.38	0.05	-6.99	0.000


*Est., estimated parameter; *SE*, standard error; Est./*SE*, ratio of the parameter estimate to the standard error; *p*, significance; regressions coefficients weighted on the dummy-coded group variable (0 = trainees; 1 = graduates); STDYX Standardization.*

In the next step, we tested whether there were group differences in the development of goal orientation in the group of graduates (see also **Tables 7**, **8**). Therefore, we calculated the neighbor change models without the group of trainees and integrated a new dummy-coded group variable (0 = graduates in vocational training, 1 = graduates at university). For mastery-approach goals, the model fit was satisfactory (χ^2^ = 413.89, *df* = 236, *p* < 0.01; CFI = 0.953; RMSEA = 0.044), and there were no significant differences in the regression coefficients at Time 1 (γ_Time1_ = 0.07, *p* = 0.27) or in the difference variables for *diff_12* (γ_diff_12_ = -0.01, *p* = 0.95) or *diff_23* (γ_diff_23_ = 0.12, *p* = 0.08).

**Table 7:** Latent means, regression coefficients, and covariances for the latent change model for mastery-approach goals within the group of graduates.

	Est.	*SE*	Est./*SE*	*p*
**Means**				
*M*_Time_ *1*	3.30	0.32	10.35	0.000
*M*_diff_12_	-0.09	0.16	-0.57	0.572
*M*_diff_23_	0.51	0.16	3.30	0.001
**Regression coefficients (β)**				
γ_Time1_	0.07	0.06	1.10	0.270
γ_diff_12_	-0.01	0.08	-0.07	0.948
γ_diff_23_	0.12	0.07	1.77	0.077
**Covariances**				
*Time 1* with *diff_12*	-0.37	0.08	-4.92	0.000
*Time 1* with *diff_23*	-0.19	0.08	-2.52	0.012
*diff_12* with *diff_23*	-0.31	0.09	-3.52	0.000


*Est., estimated parameter; *SE*, standard error; Est./*SE*, ratio of the parameter estimate to the standard error; *p*, significance; regressions coefficients weighted on the dummy-coded group variable (0 = graduates in vocational training; 1 = graduates at university); STDYX Standardization.*

**Table 8:** Latent means, regression coefficients, and covariances for the latent change model for performance-approach goals within the group of graduates.

	Est.	*SE*	Est./*SE*	*p*
**Means**				
*M*_Time_ *1*	3.46	0.27	12.65	0.000
*M*_diff_12_	-0.44	0.16	-2.77	0.006
*M*_diff_23_	0.25	0.12	2.03	0.042
**Regression coefficients (β)**				
γ_Time1_	-0.01	0.06	-0.22	0.832
γ_diff_12_	0.01	0.08	0.13	0.894
γ_diff_23_	-0.12	0.06	-1.87	0.061
**Covariances**				
*Time 1* with *diff_12*	-0.29	0.07	-4.35	0.000
*Time 1* with *diff_23*	-0.21	0.08	-2.86	0.004
*diff_12* with *diff_23*	-0.38	0.06	-6.21	0.000


*Est., estimated parameter; *SE*, standard error; Est./*SE*, ratio of the parameter estimate to the standard error; *p*, significance; regressions coefficients weighted on the dummy-coded group variable (0 = graduates in vocational training; 1 = graduates at university); STDYX Standardization.*

The results for the model of performance-approach goals were comparable to the results for the mastery-approach goals. The model fit was acceptable (χ^2^ = 546.47, *df* = 180, *p* < 0.01; CFI = 0.942; RMSEA = 0.073). We also did not find any significant differences in the regression coefficients: γ_Time1_ = -0.03, *p* = 0.83; γ_diff_12_ = 0.01, *p* = 0.89; γ_diff_23_ = -0.12, *p* = 0.06.

In summary, in the group of graduates, we did not find any differences in the development of mastery-approach or performance-approach goals between graduates who went to university and those who attended vocational training after graduating from a higher track school.

### Latent Regression Models of the Instrument Used to Measure Stage-Environment Fit and Goal Orientation

In order to analyze associations between goal orientation and the predictor of perceived stage-environment fit between a learner and their new educational context, we estimated two latent regression models (one for mastery-approach goals and one for performance-approach goals) for the group of graduates including two additional variables that were implemented to check for more internal and more external reasons for choosing a field of study or vocational training.

**FIGURE 4:**
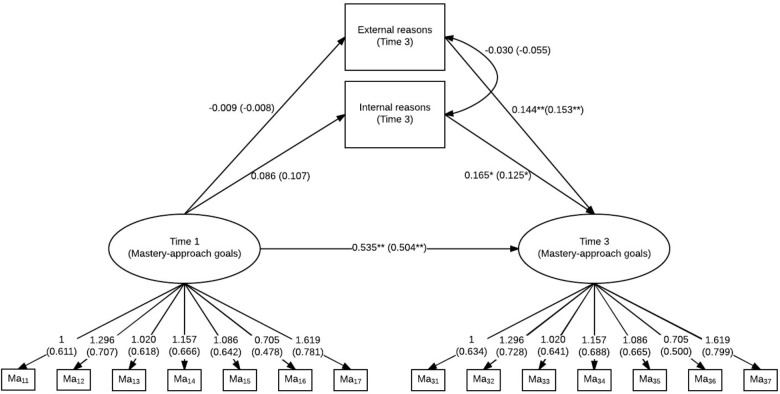
Latent regression model for mastery-approach goals (Time 1 and Time 3) and internal and external reasons for choosing a field of study or vocational training. Standardized model results (STDYX) are presented in brackets; ^∗∗^*p* < 0.01, ^∗^*p* < 0.05.

The fit for the model for mastery-approach goals was good (χ^2^ = 214.82, *df* = 119, *p* < 0.01; CFI = 0.958; RMSEA = 0.046). Mastery-approach goals at Time 3 were predicted by internal as well as external reasons (see also **Figure 4**). The regression coefficient for internal reason was slightly larger than for external reasons. In other words: Internal reasons, such as “talent” or “interest,” as well as external reasons, such as “earning opportunities” or “reputation,” both significantly predicted individual differences in the development of mastery-approach goals between Time 1 and Time 3.

**FIGURE 5:**
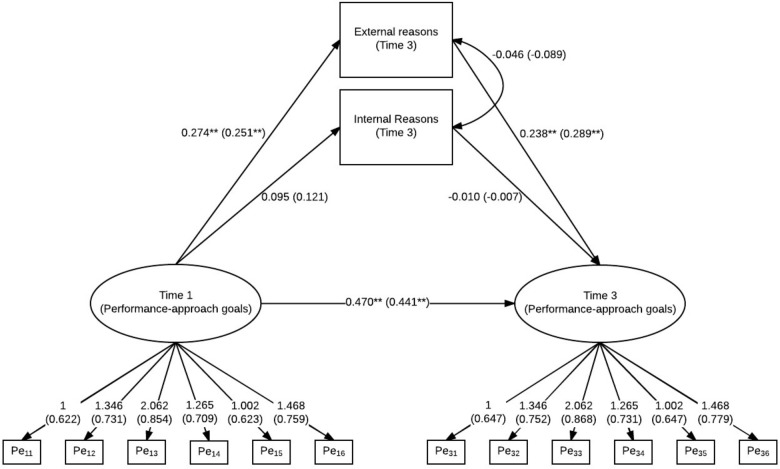
Latent regression model for performance-approach goals (Time 1 and Time 3) and internal and external reasons for choosing a field of study or vocational training. Standardized model results (STDYX) are presented in brackets; ^∗∗^*p* < 0.01, ^∗^*p* < 0.05.

The model for performance-approach goals showed acceptable fit (χ^2^ = 309.24, *df* = 92, *p* < 0.01; CFI = 0.942; RMSEA = 0.078). As expected, performance-approach goals at Time 3 were predicted by external reasons, but not by internal reasons (see also **Figure 5**). In contrast to the model of mastery-approach goals, there was also a significant regression coefficient between performance-approach goals at Time 1 and external reasons. Therefore, students indicating higher performance-approach goals at Time 1 also stated more external reasons for choosing a field of study or vocational training at Time 3 in contrast to students with lower performance-approach goals.

## Discussion

The main interest of our study was to examine the effect of the transition to a new educational context on the development of students’ and trainees’ goal orientation. We expected to find a positive impact of the transition from a higher track school to university or vocational training on mastery-approach goals. Furthermore, we tested whether there was an association between the self-reported fit of a learner’s needs and given environmental conditions as well as for changes in a learner’s goal orientation after the transition.

### Development of Mastery-Approach Goals

In accordance with our expectations, the results of the latent change analyses showed that there was an increase in mastery-approach goals for the students who graduated from a higher track school. Furthermore, it made no difference whether the graduates went on to university or began vocational training. This result is well-aligned with prior research (e.g., Meier et al., 2013; Becker et al., 2017) that also showed the positive impact of transitioning to a new educational context on the development of mastery-approach goals. In line with stage-environment fit (Eccles et al., 1993a), an increase in fit could be a likely explanation for the positive development of mastery-approach goals. In a longitudinal study, Neuenschwander, 2011, for example, found an increase in perceived fit between students’ interests and talents and their educational conditions after graduating from secondary school and starting vocational training. Also the better the fit, the more satisfied the students were with the training and their training performance. The aforementioned assumption of the correlation between the fit and positive development of education-related outcomes is well-supported by our data, which showed that graduates chose their field of study or vocational training more with regard to internal than to external reasons. The positive association between mastery-approach goals and internal reasons (interest, talent and previous experience) supported the hypothesis of a good self-reported fit between a learner and his or her environment.

Nevertheless, there was also a positive significant relation between external reasons and mastery-approach goals, which we did not expect to find. One possible explanation might be the mere fact that students have many different reasons for choosing a field of study or vocational training leads to an increase in mastery-approach goal orientation. Nevertheless, there is no empirical evidence for this assumption so far. For further research, it would be beneficial to test whether the quantity of reasons, beyond the question of whether they are intrinsic or extrinsic, have an impact on the development of mastery-approach goal orientation.

For the group of trainees, we found descriptively small changes in mastery-approach goals during the 3 years of vocational training, but in the latent change model, these changes did not appear to be significant. In our first study (Becker et al., 2017), the trainees had just begun their vocational training and reported a good fit between their needs and the new environmental conditions as well as high values of mastery-approach goals. If we assume that the perceived fit remained stable during the 3 years of vocational training, this could account for minor changes in mastery-approach goals. In this context, Roberts and Robins (2004) performed in a 4-year-longitudinal study of college students in which they showed that the person-environment fit demonstrated moderate rank-order stability and was associated with positive outcomes, such as higher self-esteem and lower neuroticism. Another explanation that is compatible with our result of the constant level of mastery-approach goals is the theory of goal structures, which postulates an impact of contextual conditions, teaching methods, and learning atmosphere on the development of goal orientation. Studies have shown that the goal structure of the learning environment itself has an influence on the motivational orientation of students (e.g., Kaplan et al., 2002; Maehr and Zusho, 2009). In particular, vocational training in previous studies focused on the development of subject-specific competencies and their practical implementation (Pätzold, 2006; Weigel et al., 2007). The goal structure of vocational training was therefore more mastery-goal oriented, thus ultimately contributing to the high level of mastery goals observed in the trainees.

### Development of Performance-Approach Goals

For graduates, we found no change in performance-approach goals between the first and second measurement points during their last 2 years in a higher track school. With regard to the association between goal structures and goal orientation, it is possible that the educational period before graduating from a higher track school is characterized by a high level of performance and competition, which leads to the stability of performance-approach goals on a high level. After the transition to university or vocational training, there was a decrease in their performance-approach goals. This result is comparable to our findings in the prior study where performance-approach goals also decreased after the transition from secondary school to vocational training (Becker et al., 2017). According to the theory of goal structures and its impact on the development of goal orientation, it is conceivable that the new learning environment is less characterized by performance-approach goal structures.

For the trainees, on the other hand, performance-approach goals decreased significantly across the 3 years of vocational training. Here again, the dominant mastery-goal-oriented structure of the learning environment (e.g., to improve competences, interests, and skills in the chosen profession) and the less dominant performance-approach structure might also explain this deterioration in performance-approach goals.

### Limitations

The current study has some limitations. First, we postulated that the changes in goal orientation would be linked to the fit between a learner’s needs and the contextual condition of a new educational situation. By analyzing the retrospective reasons that graduates gave for choosing their field of study or vocational training and their satisfaction with the new educational context, we found initial evidence for this connection. However, our analysis should not be interpreted in a causal manner. In addition, analyzing the internal and external reasons for choosing their field of study or vocational training can only be seen as a first proxy for the stage-environment fit. In our analyses, we only used self-reported data from the students, as we did not have any information about the conditions of the new educational environment. Thus, we cannot make clear assumptions about whether there is a good fit between person’s needs and the new educational environment. For further research, it would be worthwhile to integrate instruments that explicitly measure a person’s motivations and the environment’s affordances.

Further, we found a selection effect between external reasons and performance-approach goals. Students with higher values on performance-approach goals at Time 1 stated more external reasons for choosing their field of study or vocational training at Time 3, which means that the individual goal structure also may affect the choice of certain learning environments. To obtain more sustainable results, it would also be useful to measure changes in the learners’ needs and contextual conditions longitudinally.

Second, our study is not representative of the full population of graduates and trainees due to selected dropout. During the course of the BiKS longitudinal study (10 years), we had selectivity with regard to types of schools in Germany. At the end of secondary school, most of the study participants were in academic track schools (“Gymnasium/Gesamtschule”) and were not in lower track schools (“Haupt-/Realschule”). To clarify whether the results can be generalized to the entire population of this age group, research with more representative samples is needed.

Third, with regard to our sample size, we could not differentiate between different fields of study or vocational training. It is conceivable that participants differed especially with regard to learning environment and goal structure, which, in turn, could influence the development of goal orientation.

Further, our sub samples (trainees, graduates at university, graduates at vocational training) differ in their size, which could make the interpretation of the results difficult. Within the framework of structural equation modeling, the impact of different sample sizes may inflate standard errors and thus reduce the power of the examined effects. In other words, unequal sample sizes reduce the probability to detect true differences between groups. Nevertheless, in our analyses we found significant differences between these groups. Taken together, although different sample sizes reduce test power, they are not associated with systematic bias in parameter estimates for differences between these groups.

Fourth, we used data from a German longitudinal study. The German education system differs in some points from education systems in other countries. Especially with regard to vocational training, a comparable supply may not be found in most other education systems, inter alia the US. The observed effects, especially the increase of mastery-approach goal orientation, might also be due to special characteristics of German vocational training. To address this problem comparative international studies are needed. Finally, we only focused on approach-goals (mastery and performance). For more sophisticated and differentiated results, it would also be necessary to include the avoidance-goals for mastery as well as performance goals.

## Conclusion and Implications for Practice and Further Research

Our results highlight the impact of transitioning from school to a new educational context on the development of goal orientation and can offer some practical implications. Special emphasis should be placed on the increase in mastery-approach goals for graduates after the transition to university or vocational training and its correlation with measures of stage-environment fit. We assume that the increasing fit between the learners’ needs and the contextual conditions, as a consequence of the transition to a new educational system, leads to an increase in motivation. The “challenge” of the transition to a new educational context, which is predominantly chosen by own interest or talent, seems to motivate students intrinsically. Based on these results, schools should help students detect their own interests and talents, advise them in their career choices, and support them intensively in their search for a field of study or vocational training that fits their internal needs. In this context, it could also be beneficial to give students at school more opportunities to choose subjects according to their individual interest and talents. Maybe this could lead to an increase in mastery-approach goals in school. Also the consistently high levels of mastery-approach goals for peers during 3 years of vocational training showed that it is useful to motivate students to choose the next steps in their careers according to their own needs such as interest, previous experience, and talents.

For future research, it would be beneficial to plan studies concerning the goal structure of vocational training and university in detail. In particular, research should take into account whether the learning environment and teaching methods are more mastery or performance oriented. Furthermore, future longitudinal research is needed to examine whether the decrease in performance-approach goals for graduates who go on to university or vocational training is a short-term effect of their transition or whether such goals decrease (comparable to the abovementioned development during vocational training).

## Ethics Statement

At German Universities, there are rarely internal Institutional Review Boards (IRB) for Human Subject Research Protection. The German social science ethics framework consists of guidelines about good practice and ethic codes of the German professional associations and funding institutions. Our research project was funded by the German Research Foundation (DFG, Deutsche Forschungsgemeinschaft) and can therefore be seen as reviewed and approved by an equivalent review board. All study participants gave informed consent. In addition, the study was conducted in accordance with the guidelines as well as under close supervision of the Bavarian and Hesse ministries of education and culture.

## Author Contributions

SB designed the study, analyzed and interpreted the data, and wrote up the first draft of the manuscript. MP and CA designed the study, supervised the research, helped to interpret the data, and assisted in writing up the draft. All authors approved the final version of the manuscript.

## Conflict of Interest Statement

The authors declare that the research was conducted in the absence of any commercial or financial relationships that could be construed as a potential conflict of interest.

## Footnotes

1 Data of the BiKS-study are available as scientific use files (including handbooks and full documentation of all variables) on the website of the Institute for Educational Quality Improvement (IQB): http://doi.org/10.5159/IQB_BIKS_8_14_v2. Once the follow-up project BiKS^plus[8-18]^ is finished, all data will also be available as scientific use files at the same place.
