# Supplementary material for: New Challenge, New Motivation? Goal Orientation Development in Graduates of Higher Track Schools and Their Peers in Vocational Training
Source: Front Psychol. 2018 Aug 3;9:1371. doi: 10.3389/fpsyg.2018.01371 (PMC6085573; doi:10.3389/fpsyg.2018.01371)
Supplement: Supplementary file 1 [file Table_1.DOCX]

Supplementary table 1: Bivariate Correlations for Mastery-approach goals and Performance-approach-goals between the three measurement points for Graduates and Trainees.

|  | (1) | (2) | (3) | (4) | (5) | (6) |
| --- | --- | --- | --- | --- | --- | --- |
| (1) Time1 Mastery |  | .59** | .57** | .31** | .14 | .09 |
| (2) Time2 Mastery | .60** |  | .73** | .23 | .37** | .20 |
| (3) Time3 Mastery | .42** | .51** |  | .16 | .23* | .33* |
| (4) Time1 Performance | .23** | .15* | .08 |  | .68** | .57** |
| (5) Time2 Performance | .13* | .26** | .11 | .59** |  | .68** |
| (6) Time3 Performance | .06 | .08 | .19** | .47** | .60** |  |

*Note.* ** p < .01, * p < .05; correlations for the subgroup of trainees are shown above the diagonal; correlations for the subgroup of graduates are shown below the diagonal
